# Water channels in the brain and spinal cord—overview of the role of aquaporins in traumatic brain injury and traumatic spinal cord injury

**DOI:** 10.3389/fncel.2024.1414662

**Published:** 2024-05-16

**Authors:** Thea Overgaard Wichmann, Marie Hedegaard Højsager, Helle Hasager Damkier

**Affiliations:** ^1^Department of Neurosurgery, CENSE-Spine, Aarhus University Hospital, Aarhus, Denmark; ^2^Department of Biomedicine, Aarhus University, Aarhus, Denmark

**Keywords:** aquaporins, glymphatic system, traumatic brain injury, traumatic spinal cord injury, lymphatic network

## Abstract

Knowledge about the mechanisms underlying the fluid flow in the brain and spinal cord is essential for discovering the mechanisms implicated in the pathophysiology of central nervous system diseases. During recent years, research has highlighted the complexity of the fluid flow movement in the brain through a glymphatic system and a lymphatic network. Less is known about these pathways in the spinal cord. An important aspect of fluid flow movement through the glymphatic pathway is the role of water channels, especially aquaporin 1 and 4. This review provides an overview of the role of these aquaporins in brain and spinal cord, and give a short introduction to the fluid flow in brain and spinal cord during in the healthy brain and spinal cord as well as during traumatic brain and spinal cord injury. Finally, this review gives an overview of the current knowledge about the role of aquaporins in traumatic brain and spinal cord injury, highlighting some of the complexities and knowledge gaps in the field.

## 1 Introduction

Extensive research has been conducted to elucidate the mechanisms underlying the fluid flow in the brain in terms of the glymphatic system and the meningeal lymphatic network. The glymphatic astrocyte-mediated fluid transport and the lymphatic fluid transport ensure homeostasis in the central nervous system (CNS) by continuous fluid exchange. A key player in CNS physiology, and this fluid exchange, is astrocytes as they contribute to blood-brain-barrier (BBB) regulation, synaptic function, trophic and metabolic support, and a myriad of other homeostatic mechanisms (Tran et al., 2018). Still, the fluid flow in the spinal cord and some of the pathophysiological mechanisms implicated in spinal cord diseases remains elusive, notably after trauma. Trauma to the CNS is a leading cause of morbidity and mortality worldwide due to temporary or permanent impairment of brain and/or spinal cord functions (World Health Organization, 2013; Johnson and Griswold, 2017; Taylor et al., 2017; Collaborators, 2023). The pathophysiological processes following trauma to the CNS are dual with a primary injury causing CNS damage and a secondary injury exacerbating the CNS damage. This duality makes the pathophysiology of CNS traumas highly heterogenic and complex, but it also provides a window for therapeutic intervention that can limit the secondary injury. This review aims to summarize the knowledge of the glymphatic system and lymphatic network in the brain and spinal cord with focus on the role of astrocytes and their aquaporins (AQP) under normal physiological and pathophysiological conditions.

## 2 Overview of the fluid systems in brain and spinal cord

The brain and spinal cord consist of different fluid compartments that can be subdivided into four compartments: blood, interstitial fluid (or extracellular fluid), intracellular fluid, and cerebrospinal fluid (CSF). Each of these fluid compartments are connected and proper homeostasis of the CNS requires normal function of these four compartments. Contributing to the four major fluid compartments are the meningeal lymphatic vessels that are thought to participate in fluid removal from the brain and spinal cord.

### 2.1 Fluid flow in the brain parenchyma

**Figure 1:**
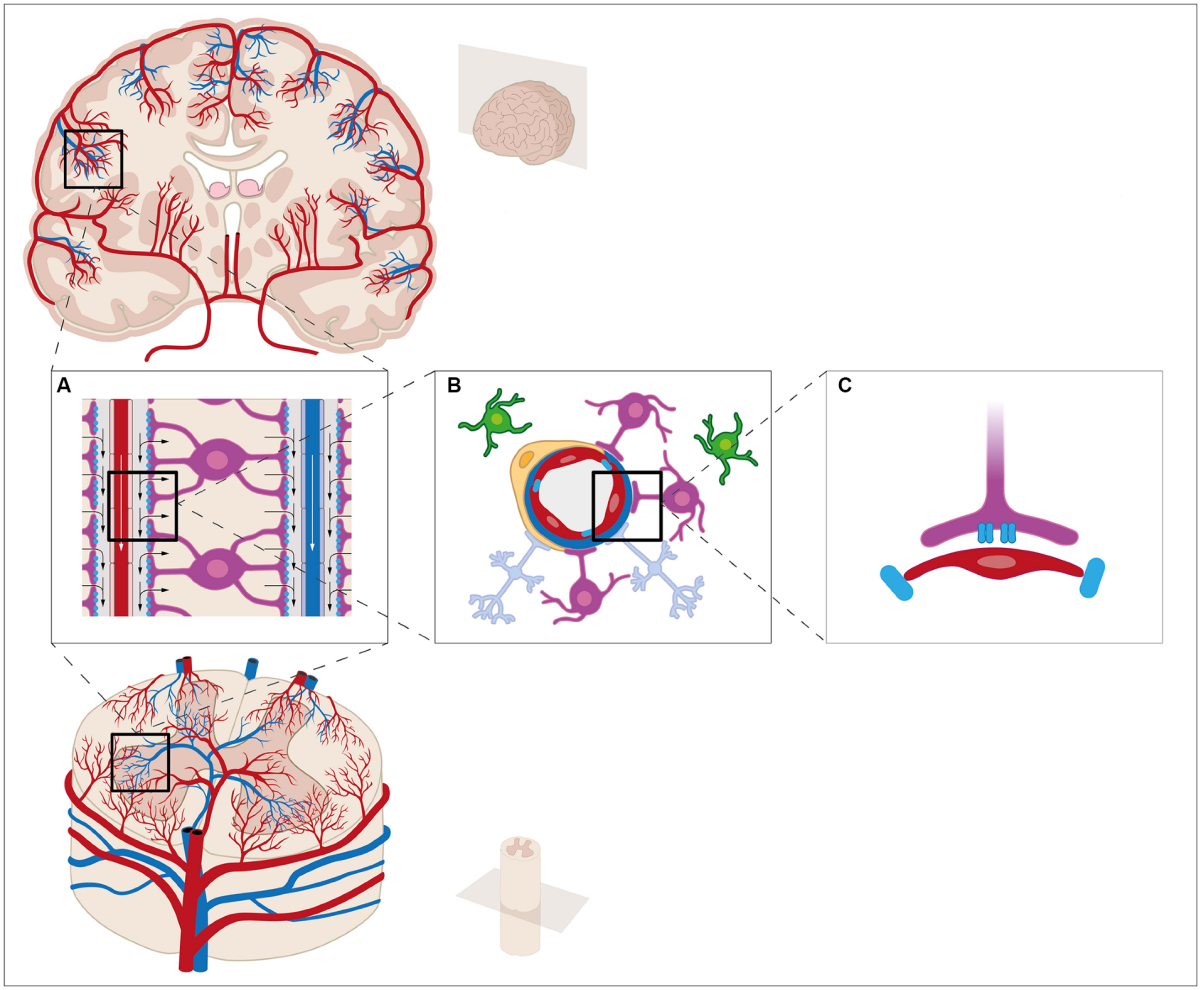
The fluid compartments of the central nervous system can be subdivided into four compartments: blood, interstitial fluid (ISF), intracellular fluid, and cerebrospinal fluid (CSF). The CSF is mainly produced by the choroid plexus in the lateral ventricles of the brain. The epithelial cells are connected by tight junctions, thereby creating the blood-CSF barrier. The brain vasculature is covered by a layer of endothelial cells connected by tight junctions, a basement membrane, and a densely packed layer of astrocytic end-feet and pericytes, thereby creating the blood–brain barrier. Surrounding the vasculature are the perivascular spaces. CSF is thought to enter from the perivascular spaces surrounding arteries **(A)** and flow into the brain parenchyma through AQP4 water channels located at the astrocytic end-feet **(B,C)**. Within the brain parenchyma, CSF disperses and intermixes with ISF and waste products. The mixture enters the perivascular spaces surrounding veins by unknown mechanisms, e.g., AQP4 water channels. From the perivascular spaces, the mixture leaves the brain parenchyma. These mechanisms have been extensively studied etc. It seems, however, reasonable to believe that similar mechanisms are present in the spinal cord.

The arterial blood supply to the brain mainly comes from the cerebral arteries that enter through the base of the skull and originate from the circle of Willis made by branches from internal carotid artery and the basilar artery which origins from the fusion of the vertebral arteries (Gray et al., 1989; Netter, 2014). The circle of Willis and the cerebral arteries are found in the subarachnoid space between the pial membrane and the arachnoid membrane. This space also contains CSF. The cerebral arteries are found on the surface of the brain, where they send branches into the parenchyma to supply the entire brain with arterial blood (Figure 1) (Gray et al., 1989). Like other organ systems, the branches become arterioles that continue into capillaries. Unlike most other organs, the capillaries of the brain are very tight and movement of solutes, water etc. is therefore restricted directly from the blood to the brain tissue. These tight capillaries are an important part of the blood–brain barrier (BBB) as endothelial cells, neurons, glia, smooth muscle cells and pericytes create the structural and functional unit of the BBB: the neurovascular unit (Profaci et al., 2020; Kugler et al., 2021). To compensate for these tight capillaries, the CSF is believed to flow along the vasculature and distribute into the neuropil (Iliff et al., 2012). The CSF flows from the surface of the brain along the arteries in the periarterial spaces (Virchow-Robin spaces, Figure 1A) (Woollam and Millen, 1955). These spaces are found along the penetrating arterioles between the basal lamina of the blood vessel and the pial basement membrane. Astrocytic endfeet attach to the parenchymal basement membrane along the arterioles (Figure 1B) (Mathiisen et al., 2010). At the level of the capillaries, the pial membrane disappears and the basement membranes of the blood vessel and parenchyma merge into a single parenchymal basement membrane leaving no apparent space for CSF (Thomsen et al., 2017). The perivascular space reappears after the capillaries in the postcapillary venules (Figure 1A) (Iliff et al., 2012). All vasculature in the CNS is innervated by sympathetic nerves and sensory nerves; however, pericytes seem to play a central role in blood flow regulation in the capillaries and postcapillary venules through neuronal activity (Hall et al., 2014).

The CSF is found most abundantly in the brain ventricles, the central canal in the spinal cord and the subarachnoid space covering the brain and spinal cord (Damkier et al., 2013). CSF is predominantly produced by the choroid plexus (Damkier et al., 2013), but extra-choroidal production has also been described. The choroid plexus is a vascularized epithelial structure located in the brain ventricles. It secretes approximately 0.5 L of CSF per day in an adult human (Damkier et al., 2013). CSF flows from the ventricles into either the central canal of the spinal cord or it exits to the subarachnoid space. The exit of CSF from the skull and vertebral canal is classically described to occur through the arachnoid granulations that protrude into the superior sagittal sinus; a venous structure located between the two dural sheets on the inside of the skull (Cushing, 1926). In this way, CSF returns to the venous system. CSF, however, can also exit through other pathways such as along the cranial and peripheral nerve sheets (Manzo et al., 1990). One pathway is along the olfactory nerves through the cribriform plate which opens into the nasal cavity (Kida et al., 1993). Here, CSF is believed to be transported with the lymph vessels that drain through the profound lymph nodes of the neck (Bradbury et al., 1981; Bradbury and Westrop, 1983).

The movement of CSF from the perivascular space around the arterioles to the neuropil and again from the neuropil to the perivascular space around the veins is described as the glymphatic system. The glymphatic system of the brain has been thoroughly reviewed previously (Jessen et al., 2015; Hladky and Barrand, 2022; Rasmussen et al., 2022). The system works as a delivery system of nutrients and metabolites for the brain tissue and a waste clearance system similar to the lymphatic system in peripheral tissue. Other groups have described a vascular basement model where the clearance of solutes is believed to occur in the peri-arterial vascular basement membrane within the tunica media, thus not involving the venules. In this model, a bidirectional movement of fluid takes place in the capillaries where inflow occurs along the glia limitans and outflow next to the endothelium (Carare et al., 2008; Arbel-Ornath et al., 2013). The mechanisms that mediate the transport of water and solutes from the perivascular space to the extracellular space and the neuropil are less well understood. The presence of aquaporins (AQPs), especially aquaporin-4 (AQP4) in the astrocytic endfeet, implies that these water channels are an entry pathway for water into the astrocytes (Wolburg et al., 2009). There are gaps between adjacent endfeet that have been estimated to be 20–50 nm in diameter, thereby enabling solutes to enter directly into the extracellular fluid (Mathiisen et al., 2010). The molecular mechanisms, however, are not resolved. A combination of diffusion and advection is believed to be involved (Koundal et al., 2020). Diffusion refers to the movement of solutes over a short distance driven by the concentration gradient and advective flow refers to flow driven by a pressure gradient generated by the pulsatile movement of the arteries. After CSF has crossed the glial membrane, CSF mixes with extracellular fluid, ions, and waste solutes in the extracellular space of the brain (Rasmussen et al., 2022). The movement of fluid in the extracellular space is not completely understood; however, studies have shown that during sleep the extracellular volume increases possibly due to movement of solutes from intra-to extracellular compartments as a consequence of especially neuronal activity (Xie et al., 2013). The glymphatic system is more effective during sleep, and it has been speculated whether the glymphatic system is under circadian control, thereby exhibiting diurnal variation. It has been shown that the influx of CSF from the perivascular space to the brain parenchyma of mice exhibits diurnal variation with increased influx during the day and decrease influx during the night. This diurnal variation has been linked to diurnal variation in localization of AQP4. The localization of AQP4 to the astrocytic endfeet increases during the day, thereby promoting CSF influx (Hablitz et al., 2020). After entering the neuropil, fluid must exit again and the amount of fluid leaving the extracellular space needs to match the inflow otherwise edema will occur. In the glymphatic system, CSF is believed to return to the perivascular spaces surrounding the veins of the brain by an unknown pathway. Unlike most other organs the inflow of arterial blood is separated from the outflow through the veins (Gray et al., 1989). The venous blood of the brain collects into two major systems: a deep and a superficial system. Most venous blood exits through the base of the skull through the internal jugular vein or through the veins in the spinal cord (Gray et al., 1989). Another minor pathway is through the skull to the superficial scalp and facial veins via the diploic veins (Netter, 2014). The veins of the CNS are characteristically devoid of valves and venous blood pressure is constantly kept low. Similar to the inflow of CSF, the molecular mechanisms driving the outflow from the extracellular space to the perivascular space at the venous side are not known. Possibly, the pressure generated by the inflow could mediate that extracellular fluid is driven back to the perivascular space around the low-pressure veins. The expression of AQP4 in the astrocytic endfeet is higher at the venous side compared to the arterial side (Iliff et al., 2012). On the other hand, the endfeet cover a larger area at the arterial side compared to the venous side, so it is uncertain whether there is any difference in net flow of water (McCaslin et al., 2011). Water transport through AQPs, however, always depends on a solute gradient, so this would require a higher concentration gradient of solutes in the perivascular space around the veins compared to the arterial side. An additional pathway for the outflow of CSF is suggested to be lymphatic vessels: however, the CNS does not contain the classical lymphatic vessels found in peripheral tissues *per se* (Aspelund et al., 2015; Jessen et al., 2015). Studies have shown that meningeal lymphatic vessels are present along the dural sinuses and along the lateral and basal parts of the skull, and represent vascular networks that clear CSF and ISF into the deep cervical lymph nodes (Aspelund et al., 2015; Louveau et al., 2015; Antila et al., 2017; Da Mesquita et al., 2018; Louveau et al., 2018; Ahn et al., 2019; Patel et al., 2019). Yet, fluid flow into and out of the CNS is difficult to study. Most studies utilize tracer injection into either CSF or into brain parenchyma, and then follow the tracer over time using various highly advanced imaging techniques either *in vivo* or after fixation. By opening the dura and injecting tracer into the CSF or the brain parenchyma may introduce increased pressure and CSF leak although much care is taken to avoid this in these elegant imaging studies. It would therefore be helpful to inject tracer into the bloodstream that, when transported across the blood-cerebrospinal fluid barrier, would change molecular composition so it would be possible to distinguish these two sites. Besides these invasive techniques using tracer injection, regular diffusion magnetic resonance imaging techniques can be used to study fluid flow in a non-invasive manner.

### 2.2 Fluid flow in the spinal cord parenchyma

The fluid compartments in the vertebral canal are similar to those of the skull. The arterial supply comes from segmental arteries from the large blood vessels close to the vertebral column, such as the vertebral arteries and the aorta (Gray et al., 1989). The blood vessels enter the spinal canal to make one anterior spinal artery and two posterior spinal arteries that generate a vascular pial plexus (Netter, 2014). From the surface, penetrating arterioles that continue into capillaries supply the nervous tissue of the spinal cord. These penetrating vessels are enveloped by astrocytic endfeet. As in the brain, the capillaries of the spinal cord are very tight with restricted movement of solutes and water from the blood to the spinal cord tissue. These capillaries also consist of non-fenestrated endothelial cells interconnected with tight junctions, basal laminae, pericytes, and astrocyte endfeet processes forming the blood-spinal cord barrier (BSCB) with a neurovascular unit consisting of neurons, astrocytes, pericytes, and microglia (Bartanusz et al., 2011). Like in the brain, CSF flows along the vessels in the perivascular space of the spinal cord (Brierley, 1950; Stoodley et al., 1996); however, tracer-analyses show that the tracer distribution in the spinal cord interstitium behaves differently in white and gray matter. In white matter, fluid diffuses along the myelinated white matter fibers. In gray matter, however, fluid flow has no specific orientation. Also, there seems to be little movement of flow from white to gray matter, whereas the opposite movement of tracer from gray to white matter has been observed (Liu et al., 2018). It was also shown that fluid can move from the spinal cord interstitial space into the central canal, thereby creating a flow-direction from the subarachnoid space to the central canal. In this way the central canal becomes a sink for the fluid (Stoodley et al., 1996). Compared to the fluid outflow in the brain, less is known about how fluid leaves the central canal and spinal cord. Despite similarities between the brain and spinal cord, an astrocyte-mediated glymphatic system has not yet been described, although astrocytes are well-described in spinal cord pathologies, including spinal cord injury (SCI). Furthermore, the presence of lymphatic vessels along the spinal cord has reached little attention compared to the lymphatic vessels in the brain. A network of lymphatic vessels has been described in rodents to be located superficially to the dural membrane and exit the vertebral canal along the spinal nerves into cervical, mediastinal, and renal/lumbar lymph nodes (Orr and Rows, 1907; Brierley and Field, 1948; Antila et al., 2017; Jacob et al., 2019). These lymphatic vessels are thought to vary in size along the spinal cord inversely correlated to the volume of CSF (Jacob et al., 2019). It has furthermore been suggested that the lymphatic network of the spinal cord is denser compared to the lymphatic network of the brain (Ahn et al., 2019). The function of these four compartments, and thereby the CNS homeostasis, is altered during disease. The brain and spinal cord can be affected by a variety of pathological conditions with trauma being a common condition impacting the CNS.

## 3 The pathophysiology of traumatic brain injury

The brain consists of three main parts: cerebrum, cerebellum, and brainstem. The cerebrum is composed of an inner, subcortical layer of white matter and an outer, cortical layer of gray mater. The white matter consists of myelinated axons and few neuronal cell bodies, while the gray mater consists of neuronal cell bodies, few myelinated axons, unmyelinated axons, dendrites, and synapses. Neuroglia cells are a heterogenic group of supportive non-neuronal cells that include astrocytes, microglia, oligodendrocytes, and oligodendrocyte progenitor cells (NG2). Astrocytes are the most abundant neuroglial cell found in both white and gray matter (Verkhratsky et al., 2019). The function of neuroglia cells is to preserve and restore CNS homeostasis by maintaining ionic balance, BBB integrity, synaptic activity, metabolism, and protein clearance (Kim et al., 2019). The role of neuroglia cells is therefore critical for maintaining the brain in a functional state that enables thinking, emotion, senses, memory, language, movement, and every other function of the body.

When sustaining a traumatic brain injury (TBI) varying degree of brain function impairment will occur. The pathophysiology of TBI has traditionally been divided into a primary injury and a secondary injury. The primary injury is caused by external direct mechanical insult, acceleration or deceleration forces, or penetrating objects causing focal and/or diffuse brain injury, including contusions, hemorrhages, and axonal injury (de Macedo et al., 2024). Following primary injury to the brain, activation of a highly complex cascade of secondary events evolves simultaneously and interact to aggravate the primary brain injury (Ng and Lee, 2019). These events include oxidative stress, mitochondrial dysfunction, edema, axonal degeneration, axonal demyelination, inflammation, synaptic dysfunction, protein aggregation, and cell death (Ng and Lee, 2019). Upon injury to the brain a neuroglial response is triggered. Astrocytes are activated and undergo proliferation, hypertrophy, and other morphological changes, including altered protein expression and translocation (Mira et al., 2021). Accompanying these changes are formation of an astroglial scar, disruption of the BBB and edema formation. Vasogenic edema develops due to disruption of the BBB integrity with extracellular fluid accumulation followed by cytotoxic edema where intracellular fluid accumulates (Donkin and Vink, 2010). Despite ionic accumulation, AQP4 contributes to the cytotoxic and vasogenic edema due to increased expression and translocation from the astrocytic endfeet to the whole astrocyte, thereby causing intracellular fluid accumulation and astrocyte swelling (Badaut, 2017). During TBI, microglia undergo morphological changes and become immunoreactive, which results in release of pro-and anti-inflammatory mediators (Amlerova et al., 2024). This triggers immune cell migration from the periphery to the injury site through the disrupted BBB. The inflammatory microenvironment induces oxidative stress and exacerbates excitotoxicity. Highly reactive oxygen species (ROS) and free radicals are generated and released from different sources causing DNA damage, lipid peroxidation, protein oxidation, mitochondrial dysfunction with impaired ATP synthesis, and thereby alterations in ion transport due to failure of the ATP-dependent Na^+^/K^+^ ATPase (Ng and Lee, 2019). This results in intracellular Na^+^ accumulation and depolarization of neurons resulting in glutamate release. Alterations in ion transport leads to opening of Ca^2+^ channels, which causes further neuron depolarization, ultimately resulting in further glutamate release and intracellular fluid accumulation (Zusman et al., 2020). The intracellular accumulation of Ca^2+^ triggers apoptotic signals leading to cell death and release of inflammatory mediators eventually causing inflammation (Amlerova et al., 2024). Neurons and oligodendrocytes are especially susceptible to apoptosis. When oligodendrocytes undergo apoptosis, myelin production decreases, causing axonal demyelination (Amlerova et al., 2024). Although demyelination is followed by remyelination, the inflammatory microenvironment and astroglial scar impairs remyelination (Jarrahi et al., 2020). The axonal demyelination leads to axonal dysfunction, and synaptopathy proximal and distal to the injury site (Hill et al., 2016).

## 4 The pathophysiology of traumatic spinal cord injury

The spinal cord is composed of an outer layer of white matter and inner layer of gray matter. The white matter consists of myelinated axons, while the gray matter consists of cell bodies of neurons, interneurons, neuroglia, unmyelinated axons, synapses, and dendrites. The role of the spinal cord is to serve as a conduit for motor, sensory, and autonomic control between the brain and body through ascending and descending pathways. This conduit requires highly organized and coordinated interactions between various neuronal cells and non-neuronal cells (O'Shea et al., 2017; Escartin et al., 2021). When sustaining a traumatic SCI, these cellular interactions are disrupted and disorganized leading to a plethora of pathophysiological events (Anjum et al., 2020). Analogous to TBI, the pathophysiology of SCI is divided into a primary injury and a secondary injury. The primary injury can be caused by mechanical damage due to vertebrae fractures, vertebrae dislocation and/or ligament tearing compressing the spinal cord (Rabinstein, 2018). Regardless the type of primary injury, the ascending and descending axonal pathways are damaged causing cell dysfunction and cell death. This triggers a plethora of secondary events that exacerbate the primary injury (Anwar et al., 2016; Alizadeh et al., 2019). The secondary injury has traditionally been subdivided into an acute, subacute, and chronic phase with overlapping injury events. The acute phase is characterized by vascular damage, increased cell permeability, edema, ionic deregulation, excitotoxicity, free radical formation, increased calcium influx, lipid peroxidation, and inflammation (Zrzavy et al., 2021). The neurovascular unit can be altered after SCI with disturbed integrity leading to perfusion changes and BSCB disruption (Bartanusz et al., 2011; Sandsmark et al., 2019). Damage of intramedullary arterioles and capillaries may cause hemorrhage and hypoperfusion, notably in the gray matter (Amar and Levy, 1999), resulting in hypoxia, ischemic cell death and ionic deregulation. Along with vascular damage, hypoperfusion may also be a consequence of neurogenic shock due to the loss of sympathetic innervation (Ruiz et al., 2018). Vascular damage leads to increased cell permeability and accumulation of cells, protein etc. which increases osmotic pressure and causes edema. Edema develops at the injury site expanding craniocaudally to adjacent segments leading to swelling of the spinal cord, compression of the spinal cord against the dura mater, and eventual ischemia and infarction (Nesic et al., 2006; Saadoun and Papadopoulos, 2010; Leonard et al., 2015). Similar to the events in the brain, edema in the spinal cord can be divided into vasogenic edema and cytotoxic edema. Vasogenic edema refers to accumulation of fluid in the interstitial space due to BSCB disruption, while cytotoxic edema refers to accumulation of fluid in the astrocytes (Garcia et al., 2023). The disruption of extracellular homeostasis, as well as the alteration of astrocytic functions due to micromilieu imbalance and water accumulation, worsen excitotoxicity (Leonard et al., 2015). The ischemia and edema may also trigger inflammatory responses (Anwar et al., 2016; Alizadeh et al., 2019). Although inflammation is triggered in the acute phase of SCI, several human and rodent studies have shown that non-resolving inflammation occurs in the subacute and chronic phase (Yang et al., 2004; Fleming et al., 2006; Schwab et al., 2014; Kwiecien et al., 2020; Zrzavy et al., 2021; Wichmann et al., 2022a,b). Along with the chemoattractants from the ischemic tissue, disruption of BSCB integrity is thought to facilitate immune cell infiltration from the blood to the spinal cord, and to activate resident immune cells (Jin et al., 2021). Disruption of BSCB integrity has been suggested to occur in the acute and subacute phase and expand craniocaudally beyond the injury site (Whetstone et al., 2003; Bartanusz et al., 2011). The finding of blood-borne immune cells at the injury site, suggest that the infiltration of immune cells is led from the blood to the injury site through cytokine and chemokine responses (Zrzavy et al., 2021). These immune cells adopt pro-or anti-inflammatory phenotypes (Brodin and Davis, 2017). Due to the ischemic and inflammatory micromilieu, microglia and macrophages express molecules involved in production of ROS that cause reactive injury. Nerve tissue is more resistant to ROS (Fitch and Silver, 2008) due to the role of the astrocytes; however, various functional changes of astrocytes emerge after SCI (Silver and Miller, 2004; Fitch and Silver, 2008) influencing ionic homeostasis (Escartin et al., 2021). The ionic homeostasis is altered due to ion channel defects with increased concentrations of Na^+^ and Ca^2+^ intracellularly and increased concentrations of K^+^ and Mg^2+^ extracellularly (Fan et al., 2018). In the acute and subacute phase of injury, cellular and extracellular matrix components deposit and form a scar surrounding the injury site. The scar is composed of multiple cell types; however, the scar is thought to have a lesion core consisting of different fibroblasts, microglia, neural stem/progenitor cells and immune cells, and a lesion border consisting of reactive astrocytes (Fawcett et al., 2012; Cregg et al., 2014; Clifford et al., 2023). Reactive astrocytes exhibit morphological, molecular, metabolic, and functional remodeling in response to injury resulting in gain or loss of functions, thus reactive astrocytes can adopt both beneficial and detrimental functions simultaneously (Escartin et al., 2021). During the acute and subacute phase, the scar seals the injury, thereby restricting spread of inflammation (Yang et al., 2020). The subacute phase is manifested by apoptotic signaling, axonal demyelination, Wallerian degeneration, and axonal remodeling. The altered ionic homeostasis activates apoptotic signaling pathways leading to further cell death and axonal damage. Wallerian degeneration refers to axonal damage and axon degradation resulting in retraction of the axon toward the soma (Clifford et al., 2023). The chronic phase is manifested by axonal dieback, maturation of glial scar, and formation of a cystic cavity. During the chronic phase of injury, the proliferation of reactive astrocytes gradually stops, and the astrocytes transform from reactive astrocytes to scar-forming astrocytes, resulting in maturation of the scar into a compact glial scar (Yang et al., 2020). The mature scar is believed to inhibit axonal regeneration and outgrowth though a physical barrier and an inhibitory extracellular matrix, thus resulting in further axonal dieback (Fleming et al., 2006; Huang et al., 2014; Vangansewinkel et al., 2016; Tran et al., 2018; Bradbury and Burnside, 2019). Despite axonal dieback, the scar may lead to the formation of a cystic cavity, which aggravates the injury further.

## 5 Aquaporins in the brain and spinal cord

Aquaporins (AQPs) are membrane proteins expressed by astrocytes serving as water channels for bidirectional water transport (Verkman, 2002). The role of AQPs in brain and spinal cord pathophysiology remains to be fully elucidated; however, the secondary injury events after TBI and SCI are thought to have functional consequences for AQPs (Salman et al., 2022). Although AQP1 and AQP4 are present in both the brain and spinal cord, AQPs in the brain has gained more attention when compared to research focusing on the spinal cord.

### 5.1 AQP1

**Figure 2:**
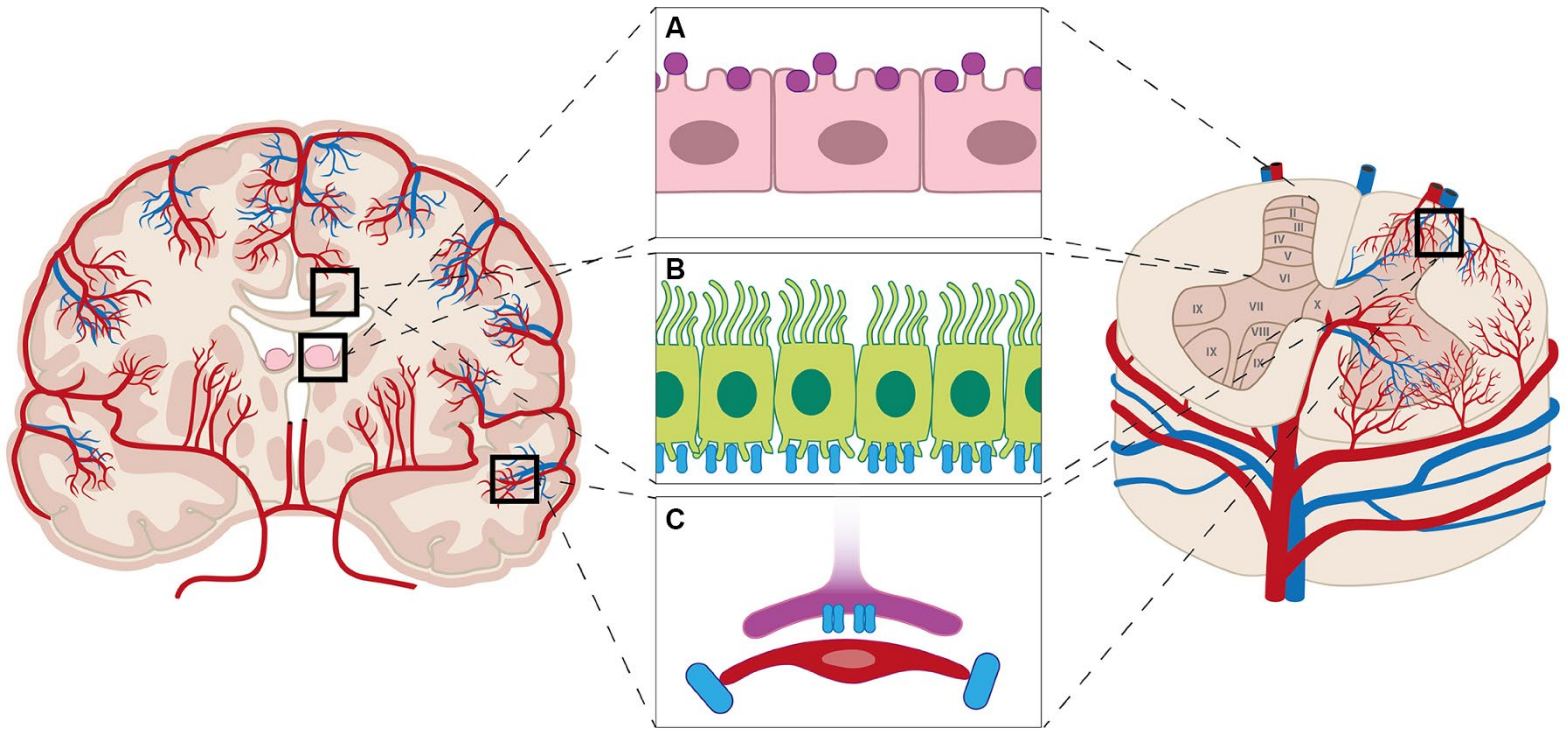
Aquaporins (AQPs) are found in the brain and spinal cord. In the brain AQP1 is expressed by the cells of choroid plexus (purple markers, **A**), while AQP1 is expressed throughout the spinal cord; however, most prominent in laminae I and II in the dorsal horn. AQP4 (blue markers) is expressed throughout the brain and spinal cord **(B)**, notably the astrocytic end-feet **(C)**, but also throughout the white and gray matter of the brain and spinal cord **(B)**.

AQP1 expression has been detected in all segments of the spinal cord and displays a uniform expression pattern throughout the spinal cord (Figure 2A) (Cushing, 1926; Oklinski et al., 2016). AQP1 is expressed in primary afferent neurons, ependymal cells, and astrocytic cell bodies (Oklinski et al., 2014). In contrast, AQP1 expression in the brain is found abundantly in the luminal membrane of the choroid plexus cells and to a smaller degree in the basolateral membrane, but not in the neuronal tissue (Nielsen et al., 1993; Oklinski et al., 2014). In the spinal cord, AQP1 is most prominent in the dorsal horn, more specifically laminae I and II as demonstrated in rat spinal cord (Nesic et al., 2008; Oklinski et al., 2014). It is believed that the labeling found in the dorsal horn is in relation to the unmyelinated sensory fibers with small diameter (Oklinski et al., 2016). AQP1 expression is furthermore found throughout the remaining gray matter along the medial border of the dorsal horn to laminae X (Oklinski et al., 2014). In white matter, AQP1 expression was found sporadically along the spinal cord, but in close relation to glial limitans associated with small penetrating arterioles (Oklinski et al., 2014). AQP1 is often found in relation to the endothelial cells in the vasculature throughout the body; however, no AQP1 labeling has been found in endothelial cells of neurovascular units or in the BBB (Oshio et al., 2006). GFAP is used as a marker for astrocytes (Li et al., 2020) and is found in high density in astrocytes in the laminae I and II, and also in fibrous astrocytes in the white matter (Kronschläger et al., 2021). As mentioned previously astrocytes have several important roles including structural, nutritional, reactive responses and regulatory aspects of the BBB (Zhou et al., 2022). Astrocytes are able to undergo large volume changes during neuronal activities on a cellular and organelle level (Zhou et al., 2022), which explains why they can impact the swelling of both brain and spinal cord tissue (Zhou et al., 2022); however, astrocytes have different specific properties based on their location in the spinal cord, e.g., astrocytes in laminae I and II are more efficient in clearing and recycling neurotransmitters when compared to astrocytes in laminae III (Kronschläger et al., 2021). A high correlation was found between Peripherin, a marker for small diameter nociceptors, and AQP1 [92% (Oklinski et al., 2016)] in the dorsal root ganglion; however, less of an overlap was found in the spinal cord (Oklinski et al., 2014). Only one isoform of AQP1 has been discovered (Zhou et al., 2022). The role of AQP1 in the spinal cord has yet to be fully unveiled. Is has been suggested that AQP1 is involved in the swelling of AQP1-expressing cells in the spinal cord after SCI (Nesic et al., 2008). AQP1 levels increased up to eight-fold in the surviving neurons post-SCI on injury sites in rats when compared to non-injury cells and persisted for up to 11 months post injury. This increase in AQP1 levels is thought to be due to excessive axonal sprouting of sensory fibers post SCI (Nesic et al., 2008). Interestingly, AQP1 expression was found in GFAP positive processes in astrocytes in injured spinal cords. They also observed a gradual decrease in AQP1 expression both caudal and rostral from the injury site, proposing a widespread impact on AQP1 expression in injured spinal cords, as well as increased AQP1 levels in scar-forming astrocytes surrounding the lesion (Nesic et al., 2008). It is still unknown what causes the increased AQP1 levels in the spinal cord; however, hypoxia may play a role in maintaining a higher level of AQP1 post injury in neurons, ependymal cells, astrocytes and sensory fibers (Nesic et al., 2008). The effects of increased levels of AQP1 are still to be discovered, but increased AQP1 levels may contribute to pathological neuronal and axonal swelling after SCI (Nesic et al., 2008) although various factors are believed to play a role in pathological swelling of the spinal cord (Oklinski et al., 2016). APQ1 has been extensively explored regarding its localization in the epithelium of the choroid plexus within the brain (Figure 2A) (Oklinski et al., 2016; Wichmann et al., 2021). More specifically, the water flux across the epithelium of the choroid plexus is believed to depend on AQP1, located in the apical membrane, and AQP1 is therefore important for CSF production (Jessen et al., 2015).

### 5.2 AQP4

AQP4 is, similarly to AQP1, expressed in all spinal cord levels in rodents and in humans (Figure 2) (Oshio et al., 2004; Nesic et al., 2006; Misu et al., 2007). Less research has been conducted on the expression of AQP4 in the spinal cord compared to brain. AQP4 is mainly found in the plasmalemma of astrocytes in both white and gray matter, in glial limiting membranes, where AQP4 is largely expressed along the entirety of the glial limitans, and might be present in ependymal cells which line the central canal in the spinal cord (Figure 2B) (Vitellaro-Zuccarello et al., 2005; Saadoun and Papadopoulos, 2010; Oklinski et al., 2014; Mader and Brimberg, 2019). The expression of AQP4 dominates in Rexed lamniae I and II within the gray matter, although it can be found throughout the gray matter in a laminar pattern (Oklinski et al., 2014, 2016; Kronschläger et al., 2021). The expression of AQP4 increases surrounding the central canal (laminae X) (Oklinski et al., 2014). AQP4 is intensely present in the astrocytic endfeet surrounding capillaries (Figure 2C) more specifically in lamina IX in the ventral horn (Oklinski et al., 2014). AQP4 can be traced along the processes in astrocytes creating a branched pattern of expression in the white matter, as well as surrounding blood vessels (Oshio et al., 2004; Oklinski et al., 2014). AQP4 is almost completely co-localized with GFAP, an astrocyte marker, within the membranes of polarized perivascular astrocytic end-feet in white-and gray matter (Vitellaro-Zuccarello et al., 2005). The area with the most co-localization is laminae I and II in the dorsal horn, in relation to glial limitans, as well as in the vicinity of the central canal (Oklinski et al., 2014). In general, the overlapping of AQP4 and GFAP is less intense in gray matter compared to the white matter astrocytes (Oklinski et al., 2014, 2016). A study showed a lack of co-labeling between glutamine-synthase and AQP4, suggesting that AQP4 is primarily located in the processes of astrocytes surrounding the capillaries and not their cell bodies (Oklinski et al., 2014).

Eight isoforms of AQP4 have been found including AQP4a (M1), AQP4b, AQP4c (M23), AQP4d, AQP4e (AQP4 Mz), AQP4f, AQP4-44, and AQP4ex (Moe et al., 2008; Rossi et al., 2011; De Bellis et al., 2014, 2017). M1 and M23 are the two most common isoforms in the CNS (Oklinski et al., 2016). AQP4 is found in glial cells in many different compartments of the brain including the olfactory system, hippocampus, thalamus, cortex, hypothalamus, subfornical organ, mesencephalon, cerebellum, and the ventricles system (Venero et al., 2001). Yet, the most dominant AQP4 expression is found in astrocytic end-feet surrounding the major fluid compartments where they form a characteristic pattern known as square arrays (Oklinski et al., 2014). Consequently, AQP4 is present at the borders between fluid compartments and parenchyma in the CNS (Saadoun and Papadopoulos, 2010) and has therefore lead to the hypothesis that AQP4 partly controls water flux in and out of the CNS both in normal and pathological conditions (Pan et al., 2022). The function of AQP4 has not been fully uncovered in the spinal cord. Nevertheless, it is believed to include regulation of local ion homeostasis, assistance in facilitating astrocyte signaling and managing of cell volume, which can play a vital role in response to SCI (Halsey et al., 2018). It has been suggested that hypertonicity impacts the AQP4 levels, leading to increased AQP4 expression and likely leading to balancing out the tonicity in the extracellular space (Nesic et al., 2008); however, altered AQP4 location might add to this balancing effect, AQP4 is believed to transport fluid to the spinal cord parenchyma from the blood or CSF and vice versa (Oklinski et al., 2014) due to its location within the spinal cord. One study described AQP4 expression in three humans with SCI. The AQP4 abundance was increased in the uninjured white matter, while sparse or undetectable at the injury site (Nesic et al., 2010). AQP4 has been found in gray and white matter in both uninjured and injured spinal cord of rodents (Oshio et al., 2004; Nesic et al., 2006). Rodent SCI studies further suggest AQP4 expression to be down-regulated early and up-regulated late and persistently (Nesic et al., 2005, 2006; Huang et al., 2019; Pan et al., 2019); however, the consequence of this is unclear. More specifically, astrocytic depletion is observed 3 days after injury at the injury site in rodents shown by lack of both AQP4 and GFAP (Nesic et al., 2010). After 7–28 days post injury, an attempt to regenerate GFAP positive cells was observed, though unclear to what extent they resemble mature resting astrocytes (Nesic et al., 2010). AQP4 levels in the newly generated GFAP positive cells were only partially restored (Nesic et al., 2010). Importantly, reduced levels of AQP4 may be present in the surviving astrocytes in SCI as well (Nesic et al., 2010). AQP4 negative astrocytes have been shown to share the same close relation to blood vessels as AQP4 positive astrocytes, as well as there is no evidence for better motor recovery in rats exhibiting low levels of AQP4 (Nesic et al., 2010). This adds to the complexity of the pathological function of AQP4 in SCI and further investigation is needed. AQP4^−/−^ mice have been used to investigate the functional role of AQP4 in SCI (Ma et al., 1997; Saadoun et al., 2008; Kimura et al., 2010; Haj-Yasein et al., 2011). Studies in AQP4^−/−^ mice confirm that water accumulates in spinal cords, thereby suggesting that AQP4 expression is required for clearance of vasogenic edema (Garcia et al., 2023). Despite the role of AQPs in SCI and SCI related conditions (Klekamp and Samii, 2002; Klekamp, 2018), AQP4 has gained increased interest due to its role in neuromyelitis optica spectrum disorder. Neuromyelitis optica spectrum disorder is an autoimmune astrocytopathy characterized by the presence of autoantibodies targeting AQP4, thereby resulting in astrocyte loss and demyelination of the CNS (Papadopoulos and Verkman, 2012).

## 6 Concluding remarks and future directions

Despite being potential targets to improve the maintenance and repair of brain and spinal cord tissue after injury, the concept of a glymphatic system and a lymphatic network of the spinal cord is a relatively under researched area compared to the brain. Despite similarities between the brain and the spinal cord regarding anatomy, physiology, and pathophysiology after trauma, all of the same theories obtained from studying the brain cannot uncritically be applied to the spinal cord, but some of the same concepts may be applied. The preponderance of studies investigating the pathophysiology of SCI are rodent studies, notably rats, as rodents are thought to resemble pathophysiological, electrophysiological, functional, and morphological features of human SCI. Although invaluable knowledge has been achieved from rodent studies, increased interest in larger animal models as pigs has emerged to increase animal-to-human translatability (Elizei and Kwon, 2017). The research in SCI pathophysiology in larger animal models and humans has increased over the past decade; however, only little information about the glymphatic system and lymphatic network along the spinal cord is available. To investigate the glymphatic system, astrocytes is a relevant focus area for further research as they contain AQPs, contribute to BBB properties by promoting BBB repair, restrict immune cell infiltration, and assist in resolution of inflammation (Sanmarco et al., 2021; Zhou et al., 2023). Furthermore, they can adopt both beneficial and detrimental functions simultaneously in response to injury. Future studies should therefore investigate different molecular marker and functional markers along with investigating the morphology and function of AQPs in response to CNS injury in larger animal models. To investigate the lymphatic network larger animal models and humans should be used to reproduce the findings of rodent studies (Jacob et al., 2019). These investigations should be followed by investigations of the functional properties of the lymphatic vessels to clarify how these central lymphatic vessels distinguish from periphery lymphatic vessels.

## Author contributions

TO: Writing – original draft, Writing – review & editing. MH: Writing – original draft, Writing – review & editing. HH: Writing – original draft, Writing – review & editing.
